# Supplementary material for: Risk factors for sacrococcygeal pilonidal sinus: a systematic review and meta-analysis supplemented by genetic causal assessment
Source: Front Surg. 2026 Jan 7;12:1718589. doi: 10.3389/fsurg.2025.1718589 (PMC12819706; doi:10.3389/fsurg.2025.1718589)
Supplement: Supplementary file 2 [file Datasheet2.zip › Supplementary Data 2/MR_pipeline_p5e-6/leaveone_plots/prot-a-529.finngen_R12_L12_PILONIDALCYST.pdf]

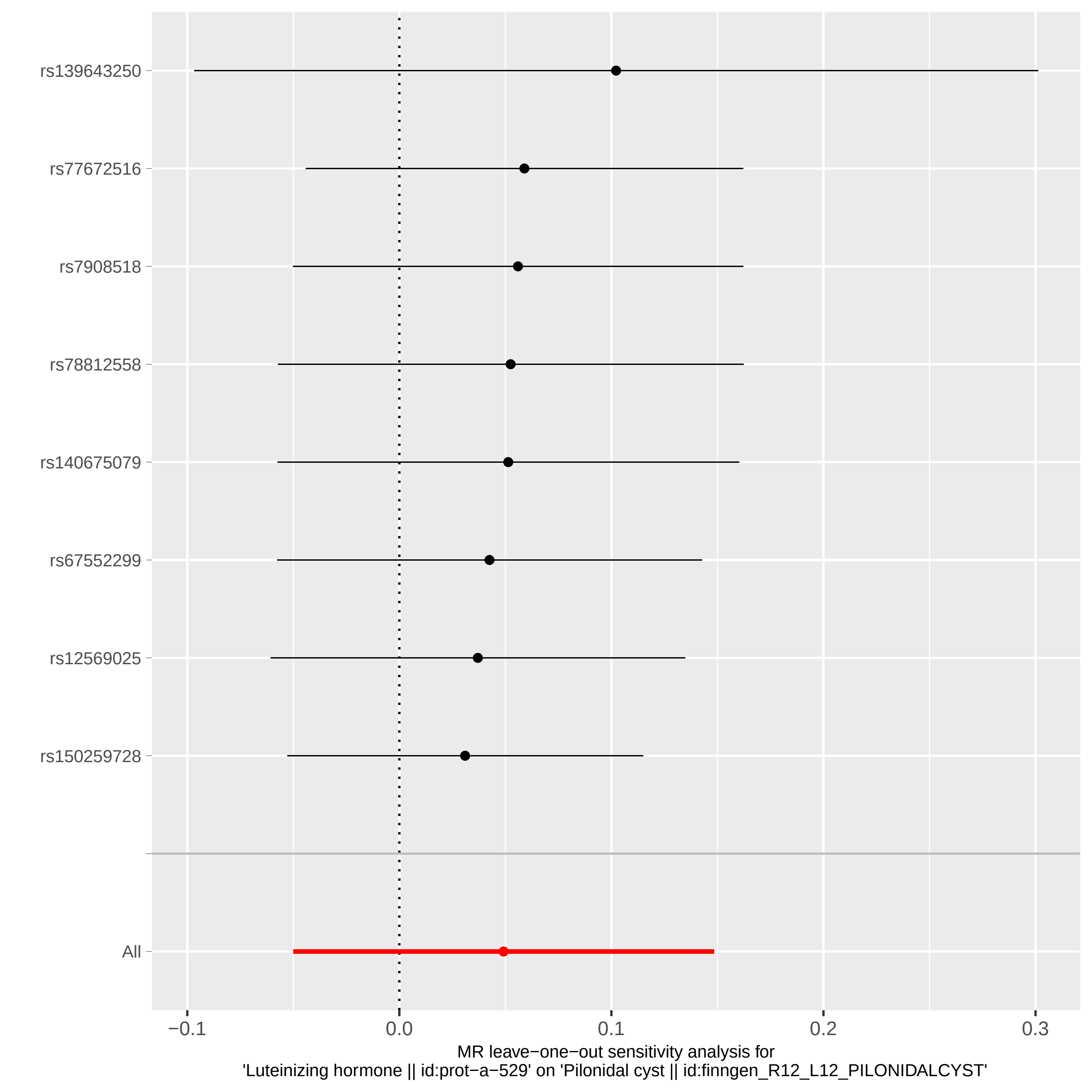
rs139643250
rs77672516
rs7908518
rs78812558
rs140675079
rs67552299
rs12569025
rs150259728
All
−0.1
0.0
0.1
0.2
0.3
MR leave−one−out sensitivity analysis for
'Luteinizing hormone || id:prot−a−529' on 'Pilonidal cyst || id:finngen_R12_L12_PILONIDALCYST'
